# Impact of extreme pre-monsoon drought on xylogenesis and intra-annual radial increments of two tree species in a tropical montane evergreen broad-leaved forest, southwest China

**DOI:** 10.1093/treephys/tpae086

**Published:** 2024-07-20

**Authors:** Ya-Nan Liu, Ze-Xin Fan, You-Xing Lin, Arisa Kaewmano, Xiao-Lian Wei, Pei-Li Fu, Jussi Grießinger, Achim Bräuning

**Affiliations:** CAS Key Laboratory of Tropical Forest Ecology, Xishuangbanna Tropical Botanical Garden, Chinese Academy of Sciences, Mengla, Yunnan 666303, China; College of Life Sciences, University of Chinese Academy of Sciences, Beijing 100049, China; CAS Key Laboratory of Tropical Forest Ecology, Xishuangbanna Tropical Botanical Garden, Chinese Academy of Sciences, Mengla, Yunnan 666303, China; Ailaoshan Station of Subtropical Forest Ecosystem Studies, Xishuangbanna Tropical Botanical Garden, Chinese Academy of Sciences, Jingdong, Yunnan 676209, China; College of Biological and Pharmaceutical Sciences, China Three Gorges University, Yichang, Hubei 443002, China; CAS Key Laboratory of Tropical Forest Ecology, Xishuangbanna Tropical Botanical Garden, Chinese Academy of Sciences, Mengla, Yunnan 666303, China; College of Life Sciences, University of Chinese Academy of Sciences, Beijing 100049, China; CAS Key Laboratory of Tropical Forest Ecology, Xishuangbanna Tropical Botanical Garden, Chinese Academy of Sciences, Mengla, Yunnan 666303, China; College of Life Sciences, University of Chinese Academy of Sciences, Beijing 100049, China; CAS Key Laboratory of Tropical Forest Ecology, Xishuangbanna Tropical Botanical Garden, Chinese Academy of Sciences, Mengla, Yunnan 666303, China; Ailaoshan Station of Subtropical Forest Ecosystem Studies, Xishuangbanna Tropical Botanical Garden, Chinese Academy of Sciences, Jingdong, Yunnan 676209, China; Department of Environment and Biodiversity, University of Salzburg, Salzburg 5020, Austria; Institute of Geography, Friedrich-Alexander-University Erlangen-Nürnberg, Wetterkreuz, Erlangen 91058, Germany

**Keywords:** *Betula alnoides*, climate response, dendrometer, *Schima wallichii*, tropical montane forest, wood formation

## Abstract

Tropical montane evergreen broad-leaved forests cover the majority of forest areas and have high carbon storage in Xishuangbanna, southwest China. However, stem radial growth dynamics and their correlations with climate factors have never been analyzed in this forest type. By combining bi-weekly microcoring and high-resolution dendrometer measurements, we monitored xylogenesis and stem radius variations of the deciduous species *Betula alnoides* Buch.-Ham. ex D. Don and the evergreen species *Schima wallichii* (DC.) Korth. We analyzed the relationships between weekly climate variables prior to sampling and the enlarging zone width or wall-thickening zone width, as well as weekly radial increments and climate factors during two consecutive years (2020 to 2021) showing contrasting hydrothermal conditions in the pre-monsoon season. In the year 2020, which was characterized by a warmer and drier pre-monsoon season, the onset of xylogenesis and radial increments of *B. alnoides* and *S. wallichii* were delayed by three months and one month, respectively, compared with the year 2021. In 2020, xylem formation and radial increments were significantly reduced for *B. alnoides*, but not for *S. wallichii*. The thickness of enlarging zone and wall-thickening zone in *S. wallichii* were positively correlated with relative humidity, and minimum and mean air temperature, but were negatively correlated with vapor pressure deficit during 2020 to 2021. The radial increments of both species showed significant positive correlations with precipitation and relative humidity, and negative correlations with vapor pressure deficit and maximum air temperature during two years. Our findings reveal that drier pre-monsoon conditions strongly delay growth initiation and reduce stem radial growth, providing deep insights to understand tree growth and carbon sequestration potential in tropical forests under a predicted increase in frequent drought events.

## Introduction

Terrestrial water storage in two-thirds of the global land area is projected to decrease under climate change scenarios by the late twenty-first century, potentially increasing the frequency of droughts (Pokhrel et al. 2021). Tropical forests account for 59% of global forest vegetation carbon and play a crucial role in climate change mitigation (Dixon et al. 1994; Bonan 2008). However, rising atmospheric vapor pressure deficit driven by global warming increased tree mortality in Australian moist tropical forests, which ultimately decreased the carbon residence duration (Bauman et al. 2022). Further studies are required to discover how forest dynamics and tree growth will respond to climatic factors under warming climate in different tropical regions globally (Rahman et al. 2019*a*). Hubau et al. (2020) found that carbon sink in Amazonian tropical forests has steadily declined over the past three decades due to increased tree mortality. Therefore, tree mortality and internal dynamics within forests are vital controls of the carbon sequestration potential (Hubau et al. 2020).

Xylem formation is a complex process underpining the dynamics of tree growth and forest productivity (Rossi et al. 2016), which comprises the majority of stem radial growth. Despite extensive tree-ring studies analyzing the variability of inter-annual radial growth and growth–climate relationships over a century in tropical regions (Worbes 2002), our knowledge of intra-annual growth dynamics for individual species remains limited due to the high biodiversity (Pumijumnong et al. 2023) and indistinct growth ring boundaries (Silva et al. 2019) in the tropics. Even by applying microcore sampling, it is often difficult to directly observe the annual cycle of xylogenesis for tree species with indistinct ring boundaries. However, high-resolution dendrometer measurements can nondestructively and continuously monitor stem radius variations with high temporal resolution, which contain irreversible radial growth of xylem and phloem as well as reversible swelling or shrinking induced by stem water status (Zweifel et al. 2000). Therefore, combining discrete xylogenesis data with continuous radial increments data may provide accurate descriptions of intra-annual xylem production and radial growth (Cruz-García et al. 2019).

In tropical monsoon regions, the intra-annual dynamics of temperature are fairly steady, but the strong seasonality of precipitation may largely impact on intra-annual wood formation and radial growth. For example, the periodicity of cambial activity and radial growth were strongly influenced by seasonal precipitation in South America (Callado et al. 2013). In Indonesia, the cambium cells were consistently divided into expanding xylem cells throughout both the rainy season and the dry season with continuous precipitation (Rahman et al. 2019*b*). Additionally, observations of the tropical tree species *Parkia nitida* and *Parkia velutina* in a tropical rainforest in French Guiana revealed that cambial activity primarily depended on seasonal precipitation and that leaf phenology was closely correlated with xylogenesis (Morel et al. 2015). So far, investigations of intra-annual xylem formation and radial growth have been scarce in southwest China. Previous studies suggested that intra-annual stem radial growth was positively correlated with precipitation and relative humidity in a tropical karst forest and a tropical ravine rainforest (Hu and Fan 2016; Kaewmano et al. 2022). Furthermore, the radial growth of pine species was mostly limited by moisture availability during the early growing season (Bi et al. 2020; Yang et al. 2022), and vapor pressure deficit negatively impacted the radial growth of *Toona ciliata* during the dry season and the dry-to-wet transition season in southwest China (Sharma et al. 2022). Consequently, water stress during the pre-monsoon season might be a key determinant limiting stem radial growth in this region. Tropical montane evergreen broad-leaved forests are the dominant vegetation type in Xishuangbanna, southwest China (Zhu et al. 2006). Here, it is of great significance to explore the intra-annual dynamics of xylem growth and stem radial increments under extreme pre-monsoon drought, as well as to analyze how radial growth responds to climatic variables.

*Betula alnoides* Buch.-Ham. ex D. Don and *Schima wallichii* (DC.) Korth. are common tree species in the tropical montane evergreen broad-leaved forest of Xishuangbanna. *Betula alnoides* is a fast-growing deciduous tree species mainly inhabiting southeast Asia and southern China (Wang et al. 2019). *Schima wallichii* is an evergreen tree species mostly distributed in the tropical forests of the north-east Himalayan region (Khanduri et al. 2013). In this study, we monitored intra-annual wood formation and radial increments of *B. alnoides* and *S. wallichii* over two years with different hydrothermal conditions during the pre-monsoon season by combining micro-sampling and dendrometer measurements. Our objectives were (i) to compare the intra-annual patterns (onset, cessation, duration, annual growth, and maximum growth rates) of the xylogenesis and radial increments for these two species during two climatologically contrasting years, and (ii) to identify the climatic factors influencing stem radial increments of *B. alnoides* and *S. wallichii* in a tropical montane evergreen broad-leaved forest. We hypothesized that (i) growth onset of both species might be delayed during drier pre-monsoon year, especially for earlier growth onset tree species, and (ii) moisture availability may dramatically impact on the radial increments of two species.

## Materials and methods

### Study site

The study was conducted in a tropical montane evergreen broad-leaved forest (21.65°N, 101.47°E, 1473.6 m a.s.l.) located in Mengla County, Dai Autonomous Prefecture of Xishuangbanna in Yunnan Province, southwest China. The study site is dominated by evergreen tree species from the families Fagaceae, Euphorbiaceae, Theaceae and Lauraceae (Zhu et al. 2006). According to climate parameters derived from Xishuangbanna Station for Tropical Rain Forest Ecosystem Studies in Xishuangbanna Tropical Botanical Garden (21.92°N, 101.27°E, 580 m a.s.l.), which is about 36 km away from the study site, mean annual air temperature was 21.9 °C and mean annual precipitation was 1484 mm during the period 1963 to 2019. Influenced by the tropical monsoon climate, around 84% of annual rainfall occurs during the rainy summer monsoon season (May to October), and only about 16% occurs during the dry season (November to April) (see Fig. S1 available as Supplementary data at *Tree Physiology* Online).

### Monitoring of xylem formation and stem radius increments

**Figure 1:**
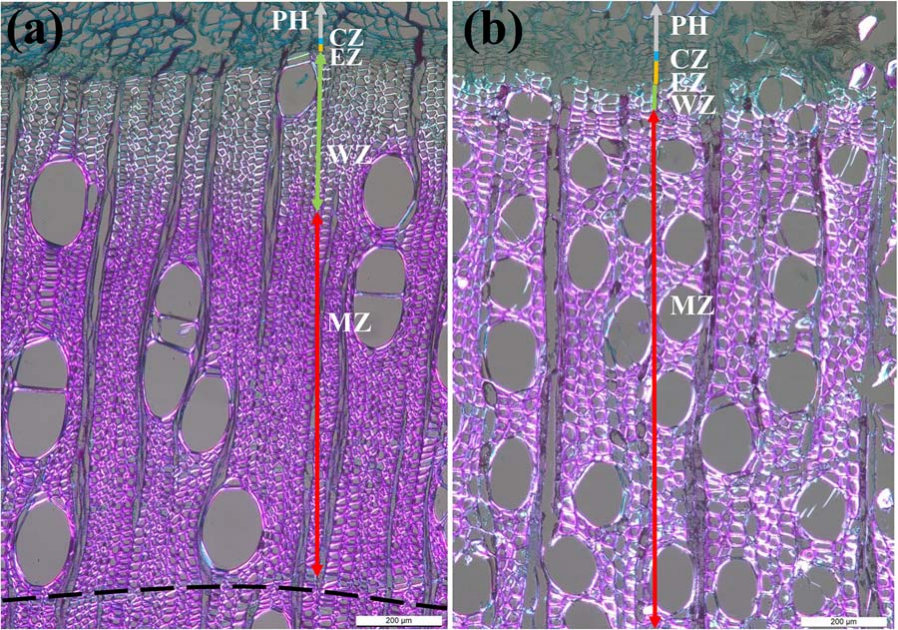
Wood anatomical structure of (a) *B. alnoides* and (b) *S. wallichii* under polarized light. PH, phloem; CZ, cambial zone; EZ, enlarging zone; WZ, wall-thickening zone; MZ, mature zone; dashed line in (a) indicates the ring boundary; bars = 200 μm.

In this study, we selected five healthy trees of the species *B. alnoides* and *S. wallichii* with an average diameter at breast height (DBH) of 32.97 ± 2.43 cm and 37.15 ± 6.80 cm, respectively. Microcores with a diameter of 2 mm and a length of 15 mm contained the intact cambium were collected at breast height using a Trephor corer (Rossi et al. 2006) in biweekly intervals from January 2020 to April 2022 to investigate the dynamics of intra-annual xylem formation. These samples were extracted spirally after removing the outer bark with a distance of approximately 5 cm among each sampling position to avoid the possible influence of local wound reactions. After collection, the microcores were placed into micro-centrifuge tubes with 50% ethanol solution and stored at 4 °C to prevent cell deterioration. Microcores were then immersed in glycerol–ethanol–water solution (mixed ratio: 10:7:3) for 2 weeks to make the samples soften. Then, these samples were dehydrated in successive ethanol solutions (70%, 80%, 85%, 95%, 95% and 100%), cleared in D-limonene and finally embedded in paraffin blocks. Transverse sections with a thickness of 12 μm were cut using a rotary microtome (RM2245, Leica, Germany), dewaxed with D-limonene, stained with safranin red and astra blue, and mounted on glass slides with Eukitt mounting medium (Sigma, Germany). Finally, these sections were analyzed under polarized light using a microscope equipped with digital camera (DM2500, Leica, Germany). After staining, cambial cells and enlarging cells were stained in blue, wall-thickening cells were stained partly blue and partly red, as well as mature and lignified xylem cells were stained in red. Cambial cells were regularly arranged in close proximity, the diameter of enlarging cells nearly doubled that of cambial cells and wall thickening cells exhibited brightness under polarized light (Fig. 1). Ring boundaries of *B. alnoides* are characterized by two to three rows of tangential parenchyma cells (Fig. 1a), while ring boundaries in *S. wallichii* are indistinct. Consequently, it was unfeasible to determine the annual ring width of *S. wallichii*. We measured the thickness of the cambial zone, the enlarging zone and the wall-thickening zone in both study species, as well as the thickness of the mature zone and the previous year’s ring of *B. alnoides* in three radial rows per picture using ImageJ software (https://imagej.nih.gov/ij/).

Four individuals of each species among the sampling microcore trees were equipped with high-resolution band dendrometers (DRL26C, EMS Brno, Czech Republic) at breast height. Stem circumference variations were automatically recorded at 10-min intervals at a resolution of 2 μm with the in-built datalogger from January 2020 to December 2021. In order to minimize the impact of shrinking and swelling within the bark, the outermost bark layers were carefully removed before installation of the dendrometer.

### Measurements of climatic factors

We installed a microclimate station to record climate variables at 10-min intervals at the study site. However, many missing data occurred because of power failure of datalogger in 2020 (see Fig. S2 available as Supplementary data at *Tree Physiology* Online). Meanwhile, we obtained daily air temperature, precipitation, and relative humidity (RH) from the meteorological station in Xishuangbanna Station for Tropical Rain Forest Ecosystem Studies, Xishuangbanna Tropical Botanical Garden (XTBG). We found close correlations of climatic factors between the two sites (see Fig. S3 available as Supplementary data at *Tree Physiology* Online), so we used climate data from XTBG for further analyses. Vapor pressure deficit (VPD) was calculated from air temperature and RH using the RHtoVPD function in R package ‘plantecophys’ (Duursma 2015).

### Data analysis

Different xylem formation stages can be accurately represented by generalized additive models (GAMs) at an intra-annual scale (Cuny et al. 2013). Hence, this study describes intra-annual changes of different xylogenesis phases for two studied species as day of year (DOY) by fitting with GAMs in R package ‘mgcv’ (Wood 2011). Because of variable annual ring width along the stem circumference, the thickness of cambial zone, enlarging zone, wall-thickening zone and mature zone of *B. alnoides* were standardized by the previous year’s ring width (Rossi et al. 2003). In addition, we defined the onset and cessation of xylogenesis as the start of the cell enlarging and the ending of the wall thickening, respectively. The growth duration was the interval between the onset and cessation of xylogenesis. Differences in xylem growth parameters (onset day, cessation day, duration and annual xylem growth) between two species and two years were compared by a t-test.

To calculate daily radial growth, we transformed the dynamics of stem circumference monitored by high-resolution dendrometer into variations of the stem radius. The stem radial variation data were corrected with temperatures monitored by the dendrometer logger using the proc_L1 function in the R package ‘treenetproc’ (Knüsel et al. 2021). The corrected stem radial variations data were further employed with the zero-growth model to extract irreversible radial growth caused by the formation of new xylem and phloem cells, and reversible shrinkage and expansion of the stem because of tree water deficit and resaturation with the R package ‘treenetproc’ (Zweifel et al. 2016; Knüsel et al. 2021). The start and end dates of radial increments for each tree were determined as the dates when stem radius variations reached 5% and 95% of total annual radial change, respectively (van der Maaten et al. 2018). Growth duration and annual radial increments were the time periods between onset and cessation of radial variations. Differences in radial increments parameters between two years and two species were tested with a t-test.

To reveal the effects of various climatic variables on wood formation and radial increments, we analyzed the relationships between weekly mean climate factors (except for weekly sum of precipitation) prior to the sampling and the thickness of enlarging zone or wall-thickening zone, as well as the correlations between weekly cumulative radial increments and concurrent climatic factors. We compared the impact of species and years on the enlarging zone width, the wall-thickening zone width and weekly radial increments (excluding zero values) by building linear mixed-effect models with individual trees as random effects and years or species as fixed effects (Chen et al. 2022). Corrected Akaike’s Information Criterion (AICc) was calculated with AICc function in R package ‘MuMIn’ to evaluate the models (Kamil 2022). The models were significantly different with AICc increments (ΔAICc) above 2. Since radial increments were significantly different in species but not years (Table 1), for the two species, we built linear mixed-effect models with the enlarging zone thickness, wall-thickening zone thickness or weekly radical increments (excluding zero values) as dependent variables, scaled climatic variables as fixed effects and individual trees nested within two years as random effects, respectively. The model is expressed as follows:


$$ \log \left(\mathrm{Y}+1\right)=\mathrm{\alpha} +\mathrm{\beta} \mathrm{X}+\mathrm{\delta} +\mathrm{\varepsilon} $$

**Table 1:** Linear mixed-effect models to evaluate the response of year (2020 and 2021) and species (*B. alnoides* and *S. wallichii*) on enlarging zone width, wall-thickening zone width and radial increments.

Models	Fixed effects	Enlarging zone width	Wall-thickening zone width	Radial increments
M1	None	0	0	0
M2	Year	1.56	0.23	0.21
M3	Species	1.71	1.00	6.95
M4	Year + species	3.28	1.35	7.08

Note: The corrected Akaike’s Information Criterion increments (ΔAICc) of each model are shown with respect to the model with the lowest score. The models were significantly different under ΔAICc > 2.

where Y represents the enlarging zone width, wall-thickening zone width or weekly radical increments of each species, X is the climate factor (precipitation, relative humidity, vapor pressure deficit, minimum air temperature, mean air temperature or maximum air temperature), α and β are the intercept and coefficient of fixed effect, and δ and ε are the variation from random effect and residual error term. The dependent variables data was log-transformed to achieve the normal distribution. All models were conducted by the lme function in the R package ‘nlme’ (Pinheiro et al. 2020). In addition, we calculated the proportion of cumulative growth on annual radial increments under different intervals of climatic factors (Tumajer et al. 2022). Subsequent data analysis and plotting were performed in R 4.0.0 (R Core Team 2020).

## Results

### Climatic conditions

**Figure 2:**
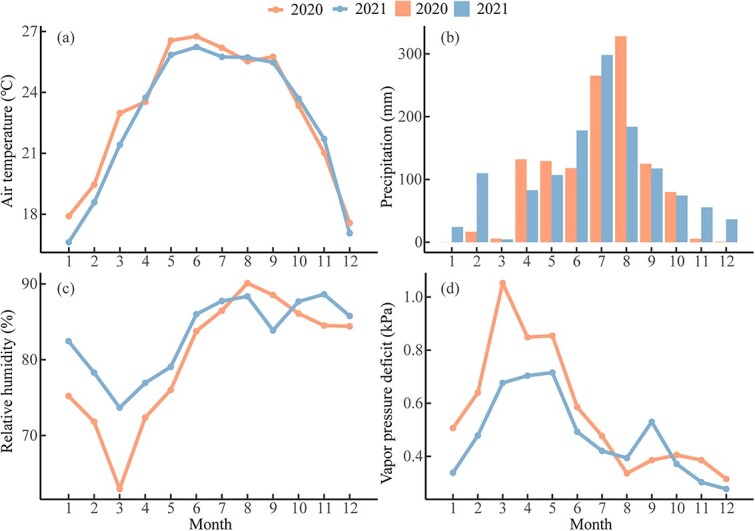
Monthly mean (a) air temperature, (b) precipitation, (c) relative humidity and (d) vapor pressure deficit in Menglun, Xishuangbanna during 2020 and 2021.

The mean air temperatures were 23 °C and 22.7 °C, as well as annual precipitation was 1206 mm and 1272 mm in 2020 and 2021, respectively (Fig. 2a and b). The climate conditions were warmer and drier than the long-term average (see Fig. S4 available as Supplementary data at *Tree Physiology* Online). The mean air temperature was higher, but precipitation was less from January to March in 2020 than in 2021 (Fig. 2a and b). The RH was lower, but VPD was higher between January and July in 2020 than 2021 (Fig. 2c and d). The year 2020 showed abnormal arid conditions during the pre-monsoon season for both RH and VPD compared with the long-term mean (see Fig. S4 available as Supplementary data at *Tree Physiology* Online). Therefore, the year 2020 had a significantly drier and warmer pre-monsoon season compared with 2021 (see Table S1 available as Supplementary data at *Tree Physiology* Online).

### Intra-annual xylem formation

**Figure 3:**
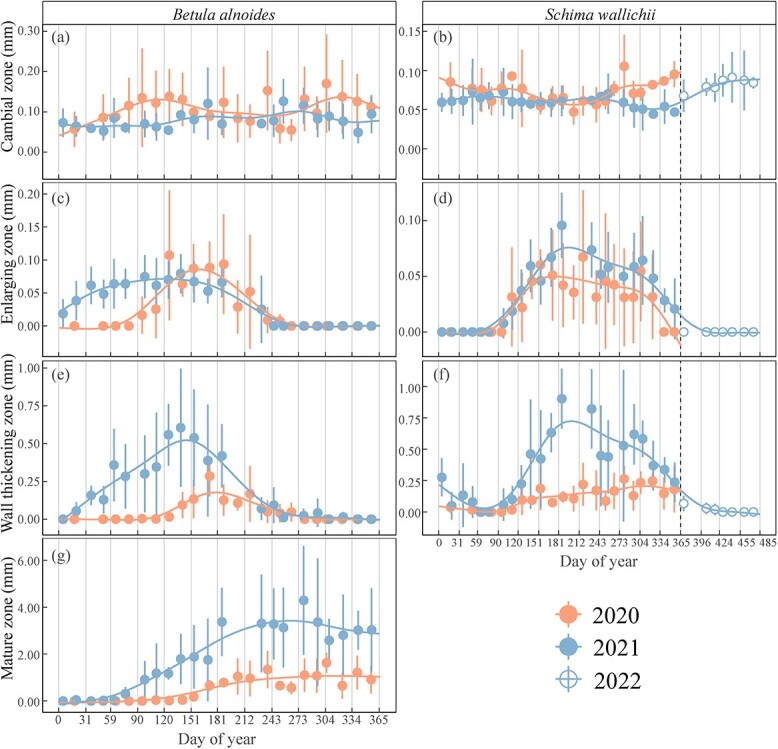
The thickness of (a, b) the cambial zone, (c, d) the enlarging zone, (e, f) the wall-thickening zone and (g) the mature zone in *B. alnoides* and *S. wallichii* during 2020 to 2021. Dots and bars represent means and standard deviations among five trees, respectively. Solid curves show fitting lines of generalized additive models based on mean values. The hollow dots with bars in b, d and f show the xylogenesis of *S. wallichii* between January and April 2022 to display an entire growing season.

The dynamics of wood formation in *B. alnoides* and *S. wallichii* varied dramatically between 2020 and 2021 (Fig. 3). In 2020, cell enlargement in *B. alnoides* started in April (DOY: 117 ± 14) and ceased in August (DOY: 238 ± 31), whereas it started in January (DOY: 17 ± 13) and stopped in September (DOY: 246 ± 22) in 2021 (Fig. 3c). The first wall thickening cells appeared in May (DOY: 135 ± 34) and January (DOY: 27 ± 9) in *B. alnoides*, whereas cell wall thickening ceased in September (DOY: 259 ± 26) and October (DOY: 274 ± 34) in 2020 and 2021, respectively (Fig. 3e). The enlarging zone in *S. wallichii* appeared in May (DOY: 146 ± 23) and stopped in November (DOY: 325 ± 33) in 2020, while it occurred in April (DOY: 117 ± 12) and ceased in December (DOY: 364 ± 8) in 2021 (Fig. 3d). However, the wall-thickening zone in *S. wallichii* ceased in early February (DOY: 34 ± 20) and later February (DOY: 50 ± 18) in 2021 and 2022, respectively (Fig. 3f).

**Figure 4:**
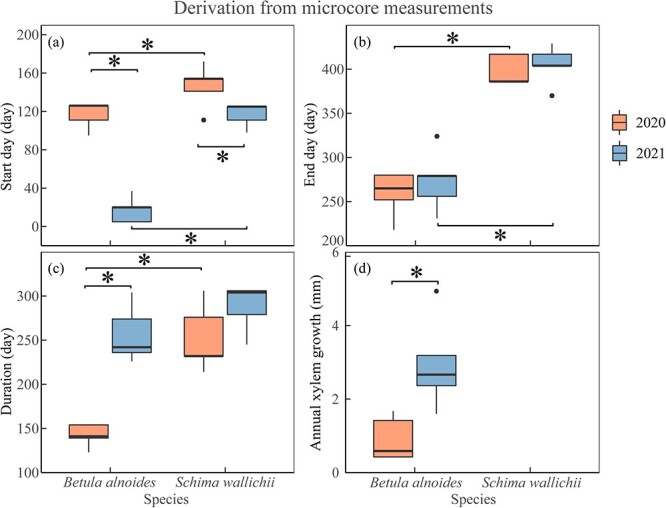
Boxplots of (a) start day, (b) end day, (c) duration and (d) annual xylem growth of *B. alnoides* and *S. wallichii* during 2020 to 2021. The data were derived from microcore measurements. The annual xylem growth of *S. wallichii* could not be measured due to its indistinct ring boundary. Asterisks indicate the significance level at *P* < 0.05.

The xylogenesis of both *B. alnoides* (*P* < 0.01) and *S. wallichii* (*P* = 0.04) started significantly later in 2020 than in 2021 (Fig. 4a). Furthermore, both onset (*P*_2020_ = 0.04; *P*_2021_ < 0.01) and cessation (*P*_2020_ < 0.01; *P*_2021_ < 0.01) of xylem formation were significantly earlier for *B. alnoides* than *S. wallichii* during the two years (Fig. 4a and b). The xylem growth duration of *B. alnoides* was significantly longer in 2021 than in 2020 (*P* < 0.01) (Fig. 4c). *Betula alnoides* grew significantly shorter than *S. wallichii* in 2020 (*P* < 0.01), while there was no significant difference in 2021 (*P* = 0.13) (Fig. 4c). In addition, annual xylem growth for *B. alnoides* was significantly lower in 2020 than in 2021 (*P* = 0.02) (Fig. 4d).

### Intra-annual radial increments

**Figure 5:**
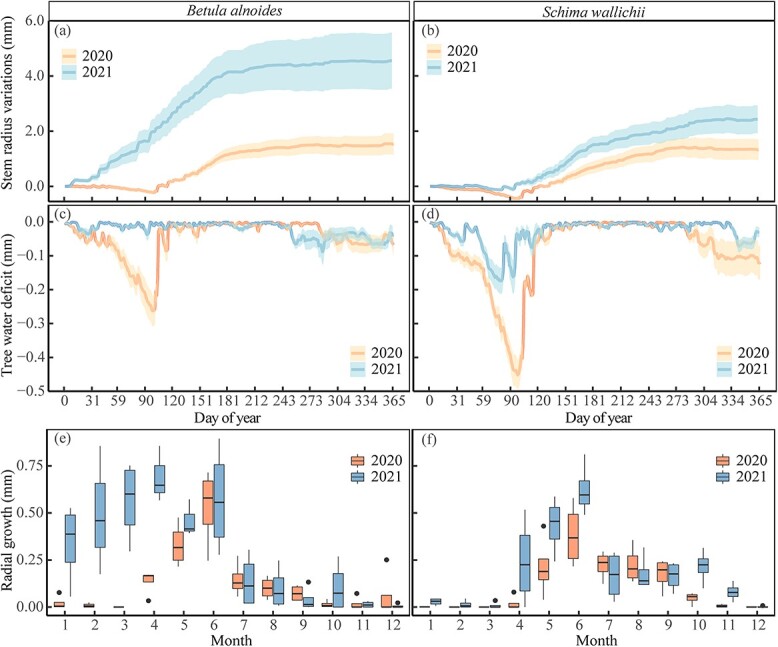
(a, b) Cumulative daily stem radius variations, (c, d) daily tree water deficit and (e, f) monthly radial growth of *B. alnoides* and *S. wallichii* during 2020 to 2021. Lines in a-d represent mean values and shaded areas represent standard errors among four trees.

Radial increments of *B. alnoides* monitored by dendrometers mainly occurred from April (DOY: 115 ± 4) to October (DOY: 283 ± 48) in 2020 and from January (DOY: 17 ± 13) to October (DOY: 274 ± 34) in 2021 (Fig. 5a and e). A distinct tree water deficit of *B. alnoides* was observed between March and April in 2020, but not in 2021 (Fig. 5c). For *S. wallichii*, radial increments appeared during May (DOY: 138 ± 13) to late September (DOY: 268 ± 8) in 2020 and during April (DOY: 110 ± 22) to the end of October (DOY: 295 ± 3) in 2021 (Fig. 5b and f). *Schima wallichii* showed a strong tree water deficit from March to April in both years; however, being much more pronounced in 2020 than 2021 (Fig. 5d).

**Figure 6:**
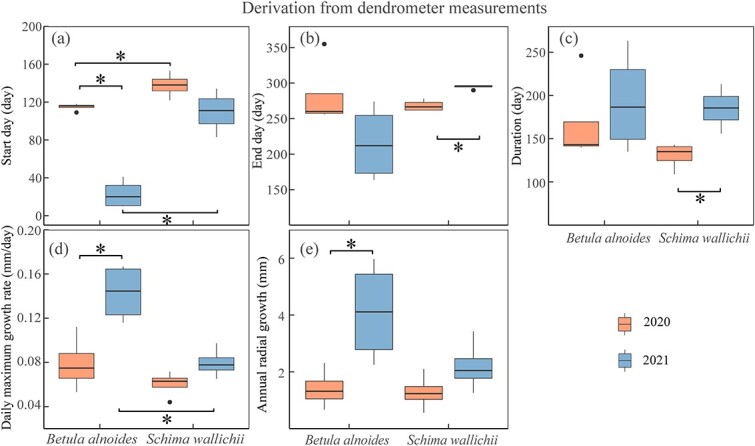
Boxplots of (a) start day, (b) end day, (c) duration, (d) daily maximum growth rate and (e) annual radial growth of *B. alnoides* and *S. wallichii* during 2020 to 2021. The data were derived from dendrometer measurements. Asterisks indicate the significance level at *P* < 0.05.

The onset of radial increments of *B. alnoides* was significantly later in 2020 than in 2021 (*P* < 0.01), while radial increments started significantly earlier in *B. alnoides* than *S. wallichii* during 2020 (*P* = 0.03) and 2021 (*P* < 0.01) (Fig. 6a). Growth cessation was significantly later (*P* < 0.01) and growth duration was significantly longer (*P* = 0.01) for *S. wallichii* in 2021 than in 2020 (Fig. 6b and c). The daily maximum growth rate (*P* = 0.01) and annual radial increments (*P* = 0.049) of *B. alnoides* were significantly lower in 2020 than in 2021 (Fig. 6d and e). Further, *B. alnoides* in 2020 showed significantly higher daily maximum growth rates compared with *S. wallichii* in 2021 (*P* = 0.01) (Fig. 6d).

### Effects of climate variables on xylogenesis and radial increments

**Figure 7:**
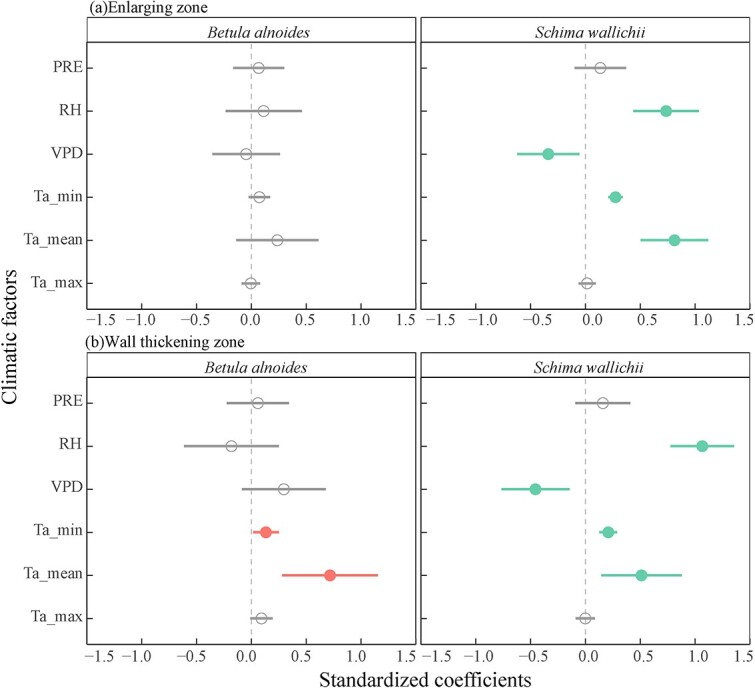
Effects of each weekly climatic factors prior to the sampling date on the thickness of (a) enlarging zone and (b) wall-thickening zone for *B. alnoides* and *S. wallichii* during 2020 to 2021. Significant levels (*P* < 0.05) were marked in closed circles. PRE, precipitation; RH, relative humidity; VPD, vapor pressure deficit; Ta_min, minimum air temperature; Ta_mean, mean air temperature; Ta_max, maximum air temperature.

Minimum and mean air temperature had a positive effect on the wall-thickening zone width of both species and the enlarging zone width of *S. wallichii* (Fig. 7). In addition, the thickness of enlarging zone and wall-thickening zone for *S. wallichii* was positively related to relative humidity and negatively related to VPD (Fig. 7).

**Figure 8:**
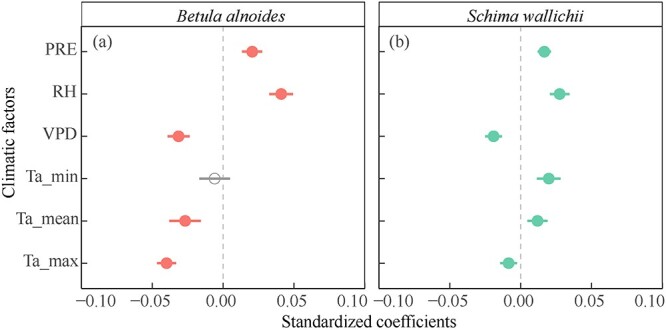
Effects of each climatic factor on weekly radial growth for (a) *B. alnoides* and (b) *S. wallichii* during 2020 to 2021. Significant levels (*P* < 0.05) were marked in closed circles. PRE, precipitation; RH, relative humidity; VPD, vapor pressure deficit; Ta_min, minimum air temperature; Ta_mean, mean air temperature; Ta_max, maximum air temperature.

Weekly radial increments of both *B. alnoides* and *S. wallichii* were significantly and positively correlated with precipitation and relative humidity, but negatively correlated with VPD and maximum air temperature (Fig. 8). The mean air temperatures showed significant negative effects on the radial increments of *B. alnoides* (Fig. 8a). However, the radial increments of *S. wallichii* were positively correlated with minimum and mean air temperatures (Fig. 8b).

**Figure 9:**
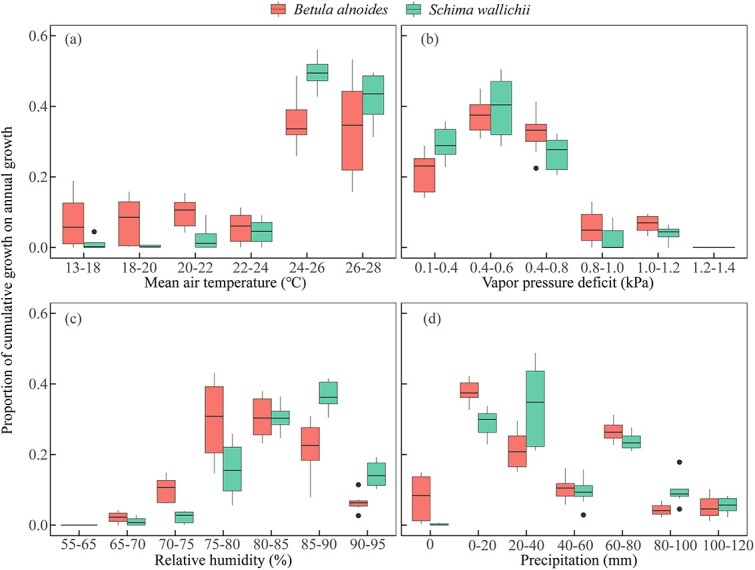
Boxplots of the proportion of cumulative growth on annual growth in specific climatic intervals for *B. alnoides* and *S. wallichii* during 2020 to 2021.

Compared with *S. wallichii*, the radial increment of *B. alnoides* occurred within a wider range of temperatures (13 to 28 °C) and precipitation (0 to 120 mm) (Fig. 9a and d). However, the radial increments of both species were highest at 24 to 28 °C or VPD below 0.8 kPa (Fig. 9a and b). The two species almost ceased their radial increments when VPD reached a level higher than 1.2 kPa or RH was below 70% (Fig. 9b and c). In addition, the radial increments of *S. wallichii* nearly stopped during the period when there was no precipitation (Fig. 9d).

### Comparison of microcoring and dendrometer methods

There were non-significant differences in the dates of growth onset between the microcoring and dendrometer methods for both species and both years. However, the dates of growth cessation and growth duration of *S. wallichii* determined by microcoring method were approximately 100 days later than those of dendrometer measurements in 2020 and 2021 (Table 2). The annual radial increments of *B. alnoides* determined by dendrometer method were around 0.5 mm and 1 mm higher than the xylem growth detected by microcoring method in 2020 and 2021, respectively (Table 2).

**Table 2:** Differences in growth phenological parameters and annual growth determined by the microcoring and dendrometer methods for *B. alnoides* and *S. wallichii* during 2020 to 2021 (mean ± SD).

Year	Species	Method	Start day (DOY)	End day (DOY)	Duration (days)	Annual growth (mm)
2020	*B. alnoides*	Microcoring	117 ± 14 a	259 ± 26 a	142 ± 13 a	0.90 ± 0.60 a
		Dendrometer	115 ± 4 a	283 ± 48 a	168 ± 52 a	1.41 ± 0.69 b
	*S. wallichii*	Microcoring	146 ± 23 a	398 ± 17 b	252 ± 38 b	
		Dendrometer	138 ± 13 a	268 ± 8 a	131 ± 15 a	1.28 ± 0.63
2021	*B. alnoides*	Microcoring	17 ± 13 a	274 ± 34 a	256 ± 32 a	2.95 ± 1.25 a
		Dendrometer	23 ± 15 a	216 ± 54 a	193 ± 59 a	4.11 ± 1.78 b
	*S. wallichii*	Microcoring	117 ± 12 a	405 ± 22 b	288 ± 27 b	
		Dendrometer	110 ± 22 a	295 ± 3 a	185 ± 24 a	2.19 ± 0.90

Different letters indicate significant difference of growth parameters monitored by microcoring and dendrometer at *P* < 0.05.

## Discussion

### Intra-annual xylem formation and radial increments

We found that both xylogenesis and radial increments of the two studied species in 2020 started three months (*B. alnoides*) and one month (*S. wallichii*) later compared with 2021 (Figs 3 and 5). Delayed growth onset may result from higher temperature, lower RH and higher VPD during the pre-monsoon season in 2020 (Fig. 2). Increased VPD could potentially reduce vegetation growth with global warming (Yuan et al. 2019). The high temperature anomaly during the pre-monsoon season in 2020 (see Fig. S4, Table S1 available as Supplementary data at *Tree Physiology* Online) may also intensify the effect of drought through altering plant carbon metabolism (Wang and Wang 2023). Similar to our results, the xylogenesis onset of *Pinus ponderosa* located in the Mojave Desert Mountain delayed two months in a year with hyper-arid spring compared with the year with wetter spring conditions (Ziaco et al. 2018). Moisture conditions in the pre-monsoon season are an important driver for the start of cambial activity. Rahman et al. (2022) observed that frequent watering during the pre-monsoon season reactivated cambial cell division of *Samanea saman* in subtropical Bangladesh. Delayed xylem formation in response to lower pre-monsoon moisture availability could be a strategy against hydraulic failure under dry conditions (Ren et al. 2015).

Both wood formation and radial increments of *B. alnoides* were significantly lower in 2020 than in 2021 (Figs 4 and 6). Annual radial increments of *S. wallichii* were also lower in 2020 than 2021, but statistically non-significant (Fig. 6). These differences between the two species might be related to their different intra-annual growth dynamics. *Betula alnoides* started growing three months earlier than *S. wallichii* in 2021. Meanwhile, we observed that *B. alnoides* began budburst and flushed new leaves between December and January, but *S. wallichii* experienced peak leaf flushing after leaf fall in March. In 2020, *B. alnoides* experienced extreme drought in the early-growing season, however the growth initiation of *S. wallichii* occurred after the drought event. Hence, the timing of drought events may determine the extent to which radial growth is constrained. Stem radial growth sharply reduced during the early-growing season drought in Eastern North America (D'Orangeville et al. 2018), while annual radial growth reduction was not observed under the European heatwave during the late-growing season in 2018 (Salómon et al. 2022). In addition, deciduous tree species have lower xylem cavitation resistance than evergreen tree species (Fu et al. 2012), which may result in greater sensitivity to water stress for the deciduous species *B. alnoides*.

### Effects of climatic factors on xylem formation and radial increments

We observed that the thickness of enlarging zone and wall-thickening zone for *S. wallichii* showed a positive response to RH, but showed a negative response to VPD (Fig. 7). Meanwhile, precipitation and RH were positively correlated with radial increments, while VPD negatively impacted the radial increments of two species (Fig. 8), indicating that stem radial increments were primarily moisture-limited in the tropical montane evergreen broad-leaved forest. Both species almost ceased their growth when no precipitation, RH below 70% or a VPD over 1.2 kPa (Fig. 9). Conversely, tree water deficits for both species occurred during the dry season (Fig. 5), especially during the warmer and drier period in 2020. Similarly, the radial growth of *Pinus kesiya* var. *langbianensis* coincided with precipitation, and tree water deficit occurred in drought in a subtropical forest in Yunnan at mid-elevations (Fan et al. 2019). In a drier site, most tree species showed stronger stem shrinkage in tropical forest (Mendivelso et al. 2016). Zweifel et al. (2021) found that stem radial growth primarily depends on water conditions and secondarily on carbon allocation. That is caused by the fact that turgor-driven cell enlarging is mostly affected by water availability, whereas the process of cell-wall thickening may rely on the availability of non-structural carbohydrates (De Micco et al. 2019). Water availability is the most important factor explaining the production of xylem cells in black spruce saplings in a greenhouse (Deslauriers et al. 2016). Correspondingly, the width of the cambial zone was positively related to monthly precipitation in evergreen moist rain forests of Ivory Coast and Thailand (Dié et al. 2012; Pumijumnong et al. 2021).

The radial increments of both species were negatively correlated to maximum air temperature, and *B. alnoides* was also restricted by mean air temperature (Fig. 8). The negative effect of temperature on radial increments is mainly linked with a higher VPD, which limited stem growth by reducing turgor pressure (Peters et al. 2023). Mendivelso et al. (2016) also revealed that high air temperature probably enhancing evapotranspiration rates and negatively affected the radial increments in two tropical dry forests. However, the wall-thickening zone width of both species was positively correlated with minimum and mean air temperature (Fig. 7). The radial increments as well as the thickness of enlarging zone for *S. wallichii* were positively related to minimum and mean air temperature (Figs 7 and 8). Similarly, Bräuning et al. (2009) found that stem radial growth of *Cedrela montana* in a humid mountain rainforest in Ecuador was positively related to temperature from January to April. Radial growth of *Quercus faginea* was limited by temperature in spring but limited by precipitation in summer in central Portugal (Vieira et al. 2022).

Though the radial increments of *B. alnoides* and *S. wallichii* responded differently to temperature, both species showed peak of growth at 24 to 28 °C (Fig. 9). Stem radial growth of three temperate broadleaved tree species peaked at 12 to 18 °C in northeastern Germany (Tumajer et al. 2022). Maximum xylem cell production of beech occurred at around 16 °C in Slovenia (Prislan et al. 2013). Moreover, balsam fir started radial growth above 9 to 10 °C in a cold and humid environment (Oogathoo et al. 2023). The temperatures during radial growth occurrence in these temperate and boreal sites were lower than those of our study.

### Comparison of microcoring and dendrometer methods

In our study, xylem formation and radial increments were simultaneously monitored by applying microcoring and dendrometer measurements. The annual radial increments obtained from dendrometer were higher compared with microcoring method (Table 2), which was consistent with the study by Kaewmano et al. (2022). Dendrometer measurements included phloem growth, though water-driven radius fluctuations were minimized with the zero-growth model (Zweifel et al. 2016). Moreover, the annual radial increments recorded by dendrometer derived from stem circumference, whereas ring width from microcoring captured radial growth in only one position (Debel et al. 2024). Consequently, both approaches inevitably show differences in annual radial growth.

Given that the wall thickening period failed to increase the number of xylem cells, growth cessation determined by microcoring data was later than dendrometer method. Meanwhile, stem radial variations were particularly susceptible to water fluctuations when the xylem growth rate was low (Deslauriers et al. 2007; Stangler et al. 2021), so the growth cessation dates monitored by dendrometer method likely deviated from the microcoring method, especially in the late growing season. Correspondingly, we found that stem radial growth of *S. wallichii* derived from dendrometer method ceased significantly earlier in 2020 than 2021 (Fig. 6), as the VPD in 2020 was higher than 2021 in September (Fig. 2). Similar to our results, Kaewmano et al. (2022) showed that the date of growth cessation monitored by dendrometers was one month earlier than that of the microcoring method. On the contrary, Debel et al. (2024) found that the growth cessation dates derived from microcoring measurements were earlier than those from dendrometer data, resulting from the fact that stem swelling increased the radial increments during the late-growing season. For coniferous trees, the application of an absolute threshold from dendrometer data, such as concrete growth rate, might be precise and unbiased for growth cessation in the southern Black Forest (Miller et al. 2022). However, it is unknown whether the absolute threshold value is fit for the identification of growth ended date in tropical forests.

Compared with microcoring, dendrometer measurements are suitable for long-term monitoring but are unable to reveal the accurate growth onset and cessation dates. Hence, models combining microcoring data with high-resolution dendrometer data are required to accurately estimate stem growth parameters. Under climate change, the frequency of drought events has gradually increased in Yunnan province during the past decades (Zhao et al. 2023). Pre-monsoon drought events caused significant growth reductions in *B. alnoides* but not in *S. wallichii* in 2020. Therefore, we should pay more attention to the earlier growth onset tree species, which are more vulnerable to extreme pre-monsoon drought events.

## Conclusion

This study simultaneously observed wood formation and stem radial variations of a deciduous tree species *B. alnoides* and an evergreen tree species *S. wallichii* during two years with differently hydrothermal pre-monsoon season in a tropical montane broad-leaved forest, southwest China. We found that the onset of xylogenesis was delayed by three months in *B. alnoides* and by one month in *S. wallichii* in 2020 (with a warmer and drier pre-monsoon season) compared with 2021. In addition, a significant growth reduction was observed in *B. alnoides* but not in *S. wallichii* during 2020. Both thickness of enlarging zone and wall-thickening zone for *S. wallichii* were positively correlated to relative humidity and negatively correlated to VPD. Weekly radial increments of both species showed positive correlations with precipitation and RH, and negative correlations to VPD and maximum air temperature, indicating that stem radial increments of these two species was mainly limited by water availability. Our results showed that tree species with an earlier growth onset were increasingly susceptible to pre-monsoon drought under climate warming conditions.

## Supplementary Material

Supplementary_materials_tpae086

## Data Availability

Data and materials supporting the findings of this study are available from the corresponding author under reasonable request.
