# Supplementary material for: Impact of extreme pre-monsoon drought on xylogenesis and intra-annual radial increments of two tree species in a tropical montane evergreen broad-leaved forest, southwest China
Source: Tree Physiol. 2024 Jul 20;44(9):tpae086. doi: 10.1093/treephys/tpae086 (PMC11387012; doi:10.1093/treephys/tpae086)
Supplement: Supplementary_materials_tpae086 [file supplementary_materials_tpae086.docx]

**Supplementary Materials**


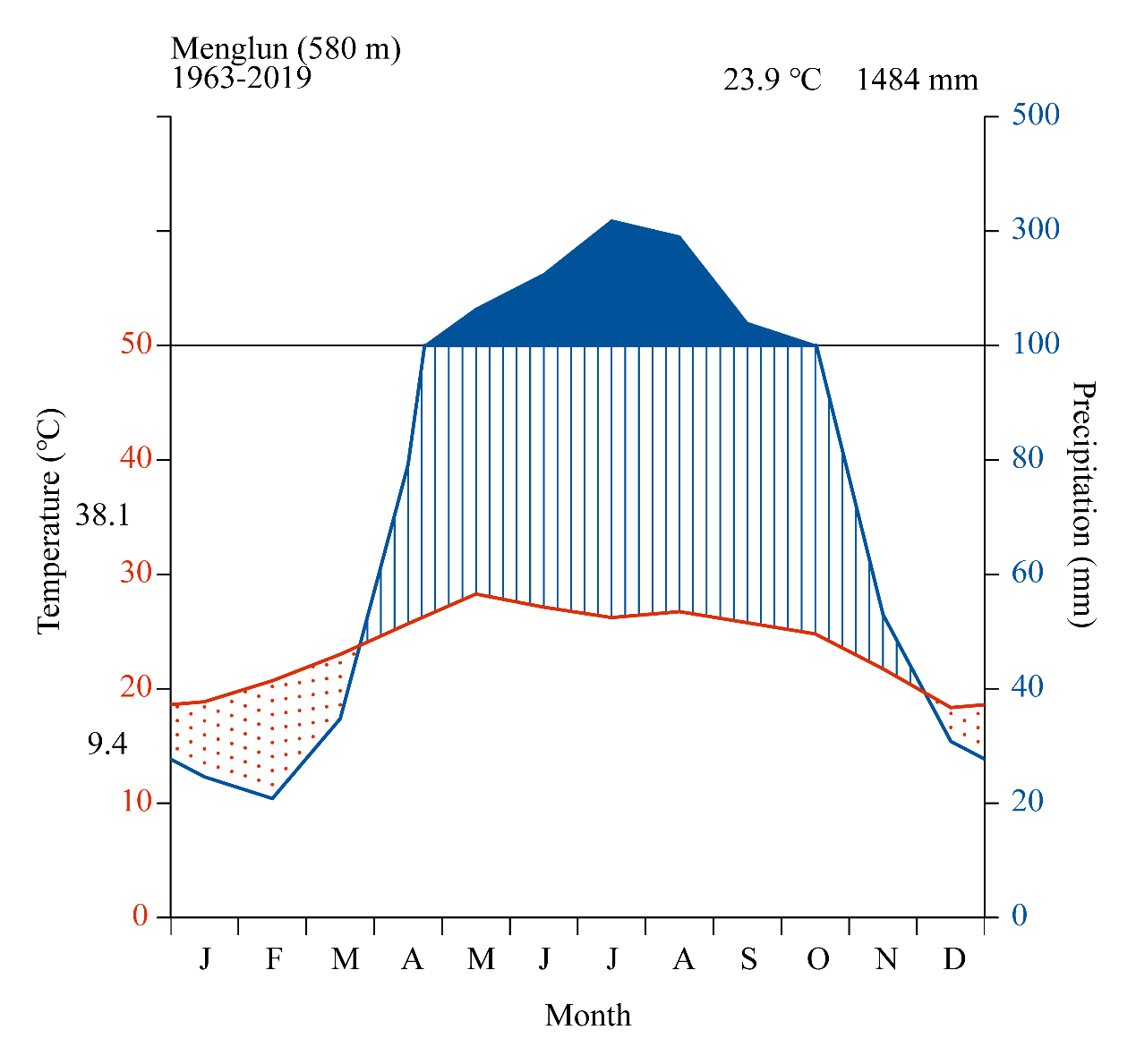


Figure S1 Walter and Lieth climograph of Menglun, Xishuangbanna, southwest China. Temperature and precipitation are represented in red and blue, respectively. Red dots, vertical blue lines, and dark blue shadow indicate dry, humid, and wet periods, respectively.


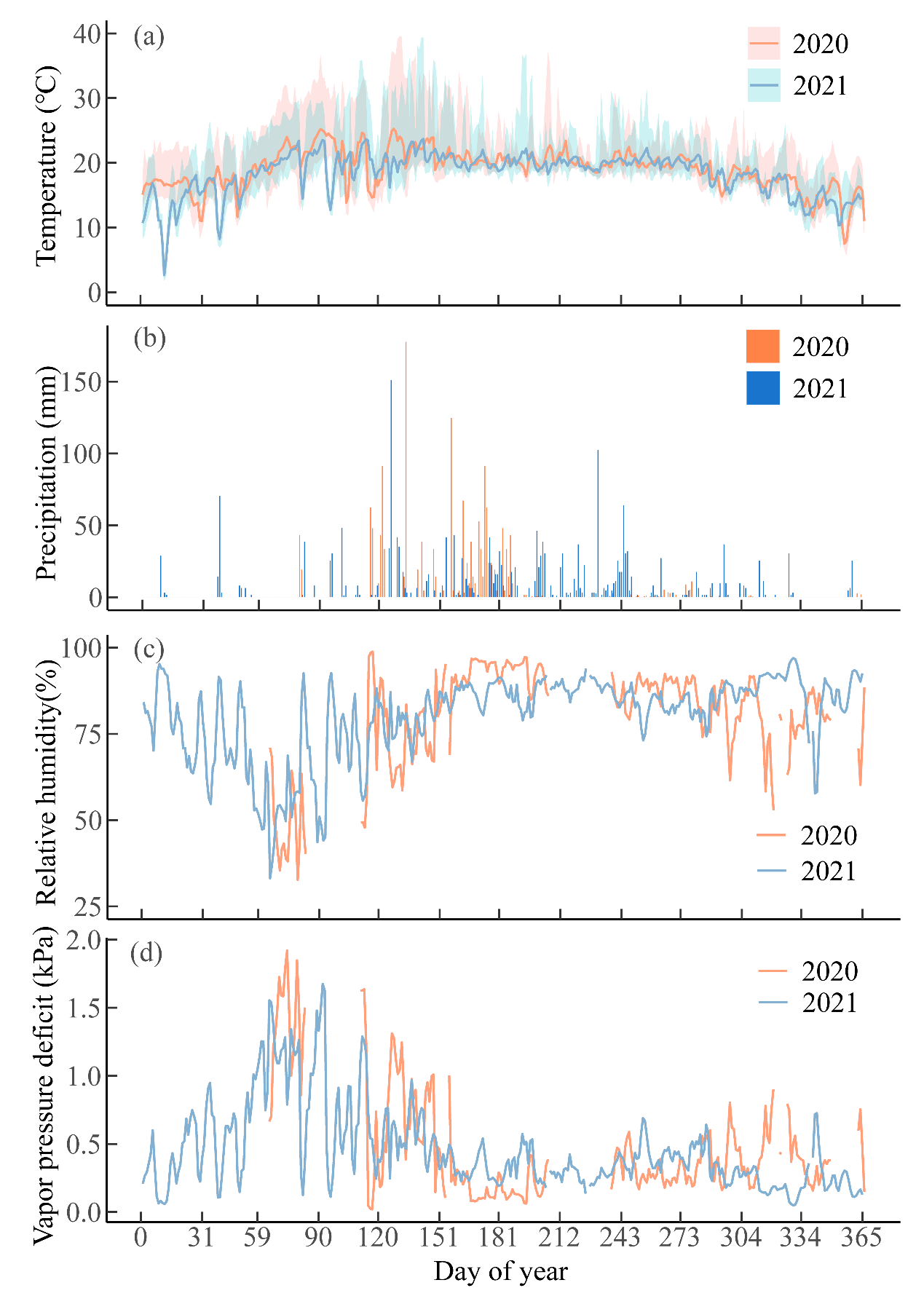


Figure S2 Daily (a) air temperature, (b) precipitation, (c) relative humidity and (d) vapor pressure deficit during 2020-2021 in Nangong Mountain, Xishuangbanna. Temperature data are obtained from dendrometer loggers, shaded areas in (a) represent daily maximum and minimum temperatures. There is a data gap in 2020 due to technical problems of the climate station.


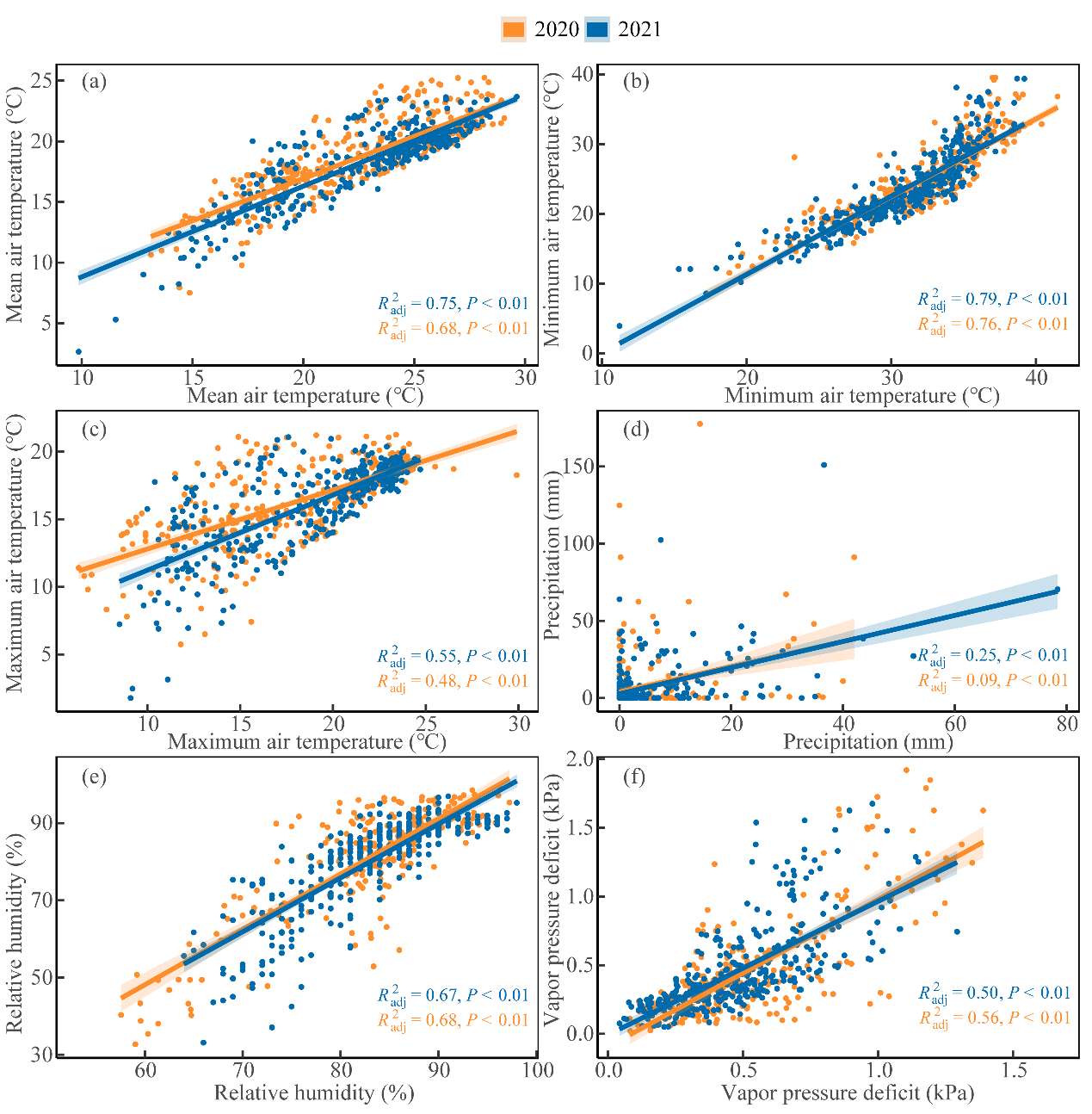


Figure S3 Linear regressions between climate variables in Nangong Mountain and in Xishuangbanna Tropical Botanical Garden. The R-square and *P* values of the linear regressions are provided in the figure.


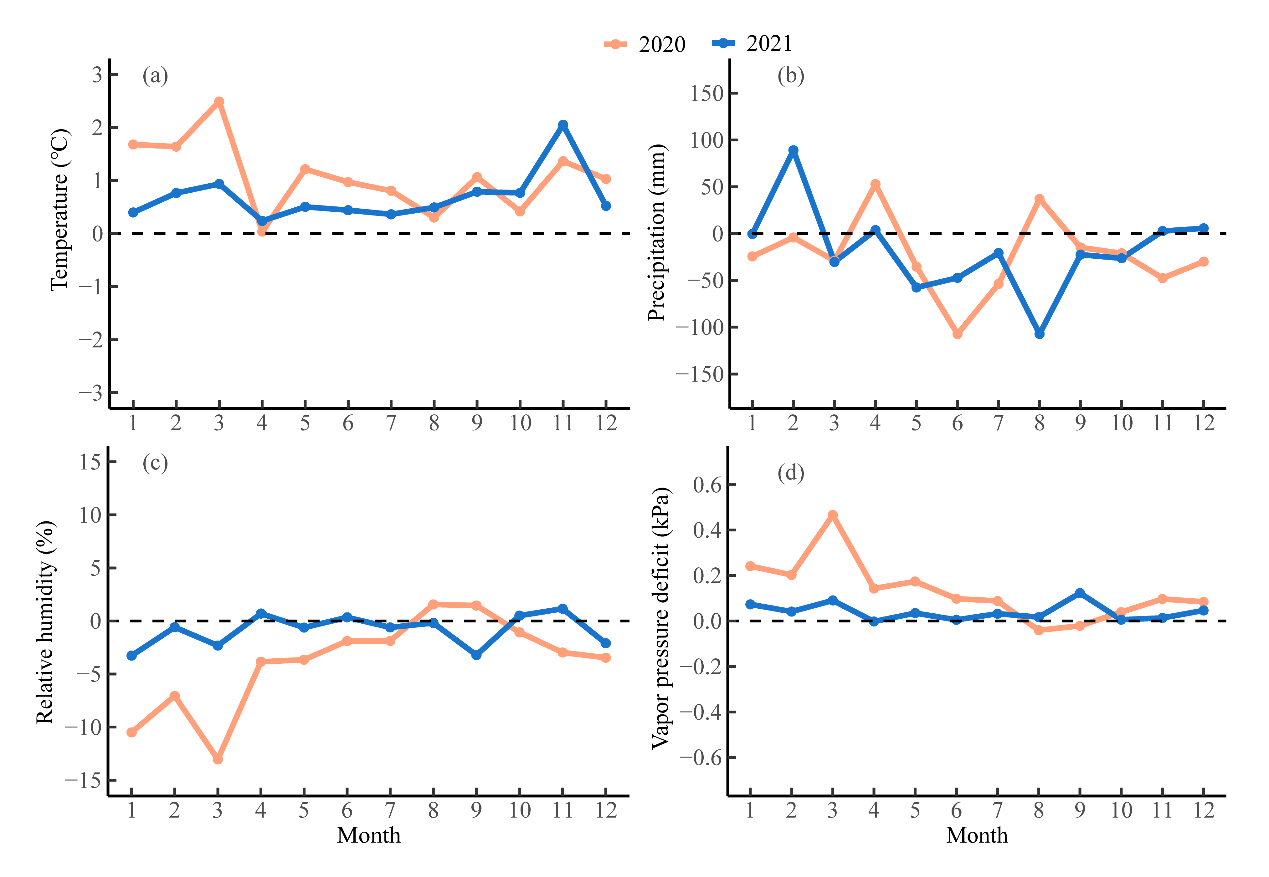


Figure S4 Anomalies of monthly mean (a) air temperature, (b) precipitation, (c) relative humidity and (d) vapor pressure deficit in 2020 and 2021 relative to the mean climate conditions during 1963-2019 in Menglun, Xishuangbanna.

Table S1 Differences in daily climate variables of the periods January-April and May-December during 2020-2021 (Mean±SD).

| Year | Month | Mean air temperature (℃) | Relative humidity (%) | Vapor pressure deficit (kPa) |
| --- | --- | --- | --- | --- |
| 2020 | January-April | 21.0 ± 3.0 a | 70.6 ± 9.2 a | 0.76 ± 0.33 a |
| 2021 | January-April | 20.1 ± 3.4 b | 77.9 ± 6.8 b | 0.55 ± 0.24 b |
| 2020 | May-December | 24.0 ± 3.4 | 85.0 ± 6.0 | 0.47 ± 0.24 |
| 2021 | May-December | 23.9 ± 3.3 | 85.9 ± 5.5 | 0.44 ± 0.22 |

**Note:** Different letters indicate significant difference of daily climate variables between two years at *P* < 0.05.
